# Comparative survey of Complementary and Alternative Medicine (CAM) attitudes, use, and information-seeking behaviour among medical students, residents & faculty

**DOI:** 10.1186/1472-6920-6-58

**Published:** 2006-12-09

**Authors:** Désirée A Lie, John Boker

**Affiliations:** 1 Department of Family Medicine, University of California, Irvine, School of Medicine, Irvine, USA

## Abstract

**Background:**

There is significant and growing national interest for introducing Complementary and Alternative Medicine (CAM) instruction into allopathic medical education. We measured CAM attitudes, use, and information-seeking behaviors as a baseline to evaluate future planned CAM instruction.

**Methods:**

Cross-sectional and longitudinal survey data on CAM attitudes, modality use, and common information resources was collected for (a) medical students (*n *= 355), (b) interns entering residencies in medical and surgical disciplines (*n *= 258), and (c) faculty from diverse health professions attending workshops on evidence-based CAM (*n *= 54). One student cohort was tracked longitudinally in their first, second and third years of training.

**Results:**

Compared to medical students and interns, faculty who teach or intend to integrate CAM into their instruction had significantly (*p *< .0005) more positive attitudes and used CAM modalities significantly (*p *< .0005) more often. Medical students followed longitudinally showed no change in their already positive attitudes. The 3 survey groups did not differ on the total number of CAM information resources they used. Each group surveyed used about two out of the five common information sources listed, with the Internet and journals most frequently cited.

**Conclusion:**

Students, interns and a selected faculty group demonstrate positive attitudes toward CAM and frequently use various CAM modalities. CAM instruction should therefore be focused on acquiring knowledge of available CAM modalities and skills to appraise evidence to appropriately advise patients on best approaches to CAM use. Trainees may benefit from exposure to a wider array of CAM information resources.

## Background

There is significant and growing national interest in the introduction and integration of CAM instruction into allopathic medical education, in part supported by recent educational funding from the National Institutes of Health. Interestingly, rates of CAM use by medical students [1] were found to be higher than reported in the US general population in 1998 [2,3] and 2004 [4]. Tracking change in learner attitudes is one strategy to document successful and effective CAM instruction. However, evaluating CAM curricular impact is complicated by the perceived heterogeneity of trainees' baseline attitudes toward CAM, per se, and the application of CAM to medical practice. A further complication is the absence of reliable, practical, and valid measures of CAM learning outcomes. Although reports have documented attitudes of medical students [5,6] and nursing, medical and pharmacy students and faculty [7], the surveys used measures that were not validated. Validation studies for two CAM attitude measures recently were reported. The first, the Integrative Medicine Attitude Questionnaire (IMAQ), compared internists attending a conference on holistic medicine to those attending an annual general professional meeting [8]. The second, the CAM Health Belief Questionnaire (CHBQ), was validated by including the IMAQ and using three cohorts (*n *= 272) of medical students at one institution [1]. The CHBQ was found to have coefficient alpha reliability = 0.75, and total CHBQ attitude scale scores positively correlated with total IMAQ scores (*r *= 0.71; *p *< .0005).

The objective of this study was to investigate and compare CAM attitudes, CAM use, and CAM information-seeking behaviours to derive priorities for CAM instruction in medical school and residency. The groups surveyed were (a) medical students, (b) medical and surgical interns at the start of their post-graduate training, and (c) faculty who teach or intend to integrate CAM into their courses or classes. We hypothesized that this selected group of faculty would have more positive attitudes, would themselves use CAM modalities at a high rate, and would use more CAM information resources than either interns or medical students. One of the two student cohorts was surveyed longitudinally in their second and third years of training to track attitude changes, if any, across the continuum of undergraduate medical training.

At the University of California, Irvine (UCI), CAM instruction for medical students at the time of the study was offered in year 1 as a 2-hour panel discussion with patients and CAM practitioners. Subsequent CAM activities were learning issues integrated into problem-based learning cases taught longitudinally across year 1. During one of 4 evidence-based medicine (EBM) classes, students were shown a variety of CAM databases and examples of CAM-related evidence and were required to perform information searches. In year 2 students interviewed at least one patient who used a CAM modality during a community preceptorship and presented the case to peers and faculty with evidence related to that CAM modality. In the third year, students received CAM instruction at noon lectures in two clerkships and were tested for their ability to counsel a patient on acupuncture and use of an herbal for osteoarthritis in the Family Medicine clerkship. Total hours of required CAM instruction across 4 years was approximately 8 hours.

The study was approved by the Institutional Review Board.

### Study sample

Student respondents included two medical student class cohorts (*n *= 355) at the University of California, Irvine (UCI), School of Medicine. There were two consecutive first-year (MS1; *n *= 170) and two consecutive second-year (MS2; *n *= 185) class cohorts. MS1 were surveyed during the first six weeks of medical school (fall of 2003 and 2004) as an in-class exercise and before exposure to any CAM instruction. One MS2 cohort was surveyed during the last six weeks of their second year (spring of 2002); and the other was surveyed midway through their second year (winter of 2002). The survey of both MS2 cohorts occurred after exposure to 3 hours of didactic CAM instruction in a required Patient Doctor course. One student cohort also was resurveyed at the end of their third year of medical school, i.e. three times in total, in year 1, 2 and 3 of medical school.

Respondents from the intern group were interns (n = 258) entering medical and surgical residencies at UCI in the academic years beginning 1 July 2002 and 2003.

Faculty respondents were faculty attending one of two workshops offered on evidence-based CAM instruction in November 2002 and October 2003 (n = 81). The faculty included nursing and physician faculty representing diverse medical disciplines that included primary care and subspecialties.

## Methods

### Measures

**Figure 1:**
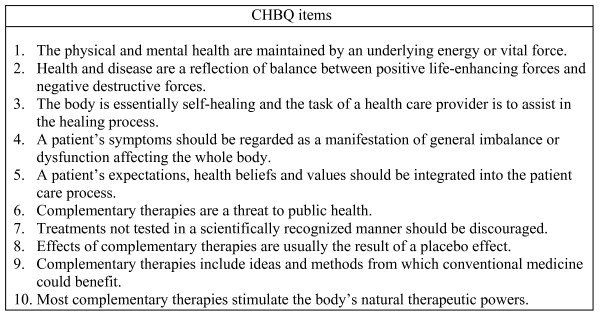
**CHBQ Items**. All items had a 7-point response scale with rating of 1 = "Absolutely Disagree" and 7 = "Absolutely Agree." (7)

The previously validated 10-item CAM Health Belief Questionnaire (CHBQ) was used to measure attitudes. Items were framed in a seven-point, Likert-type rating scale format (1 = "Absolutely Disagree," to 7 = "Absolutely Agree") (Figure 1). Responses to all CHBQ items were scored so that a higher response indicated greater endorsement and more positive attitude. CHBQ total scale scores were computed by summing across the 10 rating items. Three CHBQ items were worded negatively to minimize acquiescence response set (i.e., the tendency of respondents to reply in a consistent manner using only part of the rating scale range). Directions to the CHBQ were: "Please read and respond to each of the 10 statements below by circling the number that most agrees with your beliefs." The maximum possible score was 70 with a hypothetical midpoint of 35 (denoting neutral attitude).

The CHBQ was imbedded in a "CAM needs assessment" questionnaire. The questionnaire was constructed by adapting items from two existing instruments [6,8], and by adding items about CAM use. First, respondents' self-reported use of 14 common CAM modalities was requested. The 14 modalities were derived from categories used by the National Center for CAM [9]. Second, awareness and use of primary online and other CAM information resources (books, Internet, journals, videos and health databases) were assessed. Both of the latter item sets elicited binary ("Yes"/"No") responses.

### Data collection

Medical students were asked to anonymously and voluntarily complete the structured written questionnaire. MS1 completed the survey at the beginning of a CAM class and MS 2 completed the survey at the end of other required Patient Doctor classes. The questionnaire was administered by a staff member who was not involved in the students' instruction or evaluation. Data were collected at one sitting lasting about 20 minutes. One student cohort completed the survey a third time during a third-year clinical practice examination.

Interns were asked to complete the same written questionnaire at an orientation to residency in the first week of July by a staff member unrelated to the project. Questionnaires were completed at one sitting within 20 minutes.

Faculty attending the one-day workshops received the questionnaire at registration and were asked to complete the surveys anonymously and return them in a labelled box.

No incentives were given for completion of questionnaires.

### Data analysis

Analyses were performed using SPSS version 13 software (SPSS, Inc., Chicago, Illinois). First, descriptive statistics for all variables across all respondents were computed. Demographics of respondents (age, gender and self-identified ethnicity) were documented. Demographics for each type of respondent (student, intern and faculty) were reported in aggregate. The data for MS1 and MS2 were pooled for each level of learner. Between-group comparisons of CHBQ total scores and overall CAM modality use were made by one-way analysis of variance; significant *F*-tests were followed by pair-wise, independent *t*-tests.

## Results

The final sample included 667 respondents with 53% (n = 355) medical students, 39% (n = 258) interns and 8% (n = 54) faculty. Response rate for medical student respondents was 96.5%. Response rate for intern respondents was 100%. Response rate for faculty respondents was 60%.

### Respondents and their characteristics

50% of respondents were male. 43% of student respondents identified themselves as white compared to 40% of interns and 59% of faculty. 92% of student respondents were aged 20–29 compared to 66% of interns and 7% of faculty. 8% of students were aged over 30 years compared to 34% of interns and 93% of faculty.

### CAM modality use (Figure 2)

Faculty used a significantly higher total number of CAM modalities than either students or interns (*F *= 26.18; *p *< .0005). Students and interns did not differ in this regard. Furthermore, faculty were most likely to use each CAM modality. Across the 3 groups of respondents, massage was the most frequently used modality, followed by Spirituality and herbals. The next 3 highest rates of use occurred for meditation, chiropractic and traditional Oriental Medicine. Students and interns were similarly likely to use meditation and chiropractic but their use was lower than that of faculty.

### CHBQ scores (Figures 3 and 4)

**Figure 3:**
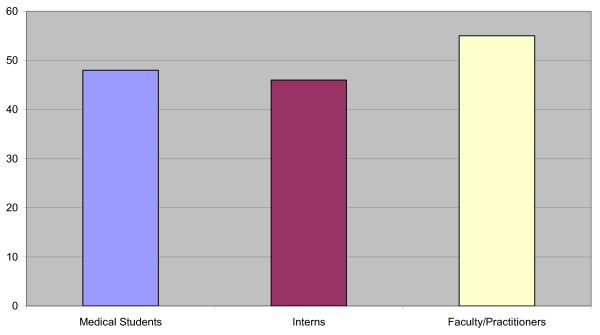
**CHBQ mean scores for students, interns and faculty**. x-axis: Respondent type; y-axis: Respondent score (%); p < .0005 faculty vs students or interns.

**Figure 4:**
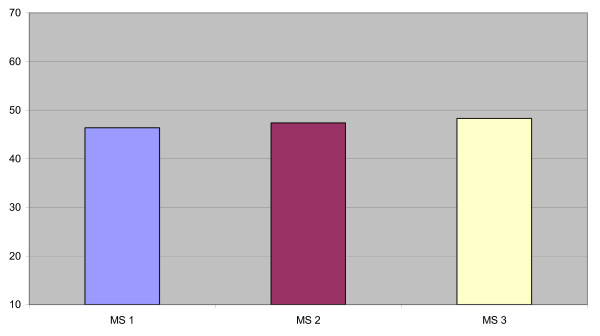
**Cohort CHBQ mean scores at three points in curriculum (students)**. No significant change in Mean CHBQ across curriculum; p = .179.

Mean scores for all 3 respondent groups exceeded (*p *< .0005) the hypothetical scale midpoint of 35 (i.e., a score representing neutral responses to CAM practice and use). Mean score was highest (*F *= 24.71; *p *< .0005) for faculty (54.5) and similar for medical students (47.8) and interns (46.2) (Figure 3). Medical students at 3 different points in their training (years 1, 2 and 3) showed similar (*p *= .205) mean CHBQ scores (46.4, 47.4, 48.3, respectively; see Figure 4) with no significant change (*p *= .179) in mean scores for the cohort followed from year 1 to 3.

### Use of CAM resources

The most commonly identified resources for CAM information for all 3 respondent groups were the Internet (75 to 80%), followed by journals (40 to 70%) and books (40 to 60%). Faculty were twice as likely to use journals as resources compared to students and interns. Of online resources used, PubMed was identified most often (75 to 85% of respondents) followed by the Cochrane Library with few identifying use of the German E Commission and Combined Health Database as resources used. Faculty were more than twice as likely to use the Cochrane library compared to interns (60% identified this resource vs. 25% of interns).

## Discussion and conclusion

We conducted a survey of attitudes toward CAM and CAM use that included rate of self-use of CAM modalities and information-seeking behaviors amongst medical students, interns and selected faculty attending CAM workshops. We used a previously validated measure, the CHBQ, to assess CAM attitudes. Not surprisingly, faculty were likely to have more positive attitudes on the CHBQ compared to interns and students. They also demonstrated higher use of CAM modalities than medical students and interns. A notable result was that student attitudes toward CAM and CAM use remained stable and positive and did not deteriorate over the course of training from year 1 to 3 as might be expected from exposure to negative attitudes toward CAM during clinical training. It is possible that our longitudinal integrated instruction maintained positive attitudes toward CAM and CAM use. It is also likely that student attitudes are relatively resilient to change during training. If so, we speculate that CAM instruction could be best directed toward increasing student knowledge of CAM modalities available in their communities and skills to access, appraise and interpret evidence on CAM use, to appropriately advise patients.

The finding of intern attitudes that are as positive as medical students is encouraging and suggests that CAM instruction in residency may be similarly directed toward increasing skill and knowledge rather than changing attitudes.

Our study has some limitations. It was conducted at one institution at which positive student attitudes have previously been demonstrated and may not be generalizable to other institutions and settings. The attitude of other faculty, to whom students are exposed, during four years of training was not elicited. Despite these limitations, our data give cause for optimism and progress in measuring the outcomes of CAM instruction. We conclude that given the stability and positive attitudes of students over the course of training, student and resident instruction in CAM could focus mainly on knowledge and skill acquisition, rather than changing attitudes toward CAM. A comparative study with other medical schools would help to elucidate possible differences in CAM instructional needs across medical schools.

## Competing interests

The author(s) declare that they have no competing interests.

## Authors' contributions

DL designed the comparative study with JB, administered the surveys, completed the literature review and background, and wrote the first draft of the manuscript with revisions by JB. JB analyzed and interpreted data and is responsible for statistical reporting. Both authors conceived the study, edited and revised the manuscript for resubmission.

## Pre-publication history

The pre-publication history for this paper can be accessed here:



## Figures and Tables

**Figure 2:**
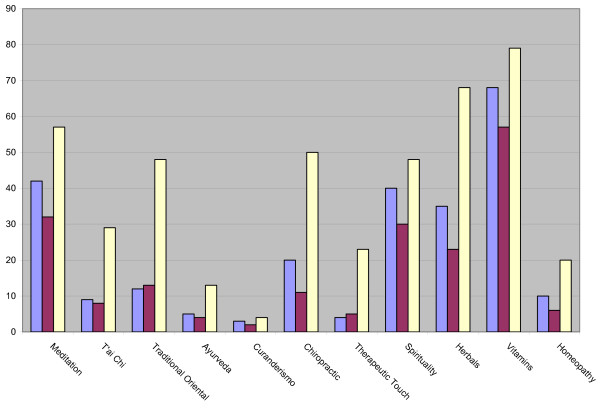
**2 – CAM modality use by students, interns, and faculty**. x-axis: CAM modalities; y-axis: Respondent use (%); For each group, from left to right the bars represent: medical students (n = 355), interns (n = 358), and faculty/practitioners (n = 54); p < .0005 faculty vs students or interns.

## References

[B1] Lie D, Boker J (2004). Development and validation of the CAM Health Belief Questionnaire (CHBQ) and CAM use and attitudes amongst medical students. BMC Med Educ.

[B2] Astin JA (1998). Why patients use alternative medicine: results of a national study. Jama.

[B3] Eisenberg DM, Davis RB, Ettner SL, Appel S, Wilkey S, Van Rompay M, Kessler RC (1998). Trends in alternative medicine use in the United States, 1990–1997: results of a follow-up national survey. Jama.

[B4] Barnes PM, Powell-Griner E, McFann K, Nahin RL (2004). Complementary and alternative medicine use among adults: United States, 2002. Adv Data.

[B5] Chez RA, Jonas WB, Crawford C (2001). A survey of medical students' opinions about complementary and alternative medicine. Am J Obstet Gynecol.

[B6] Hopper I, Cohen M (1998). Complementary therapies and the medical profession: a study of medical students' attitudes. Altern Ther Health Med.

[B7] Kreitzer MJ, Mitten D, Harris I, Shandeling J (2002). Attitudes toward CAM among medical, nursing, and pharmacy faculty and students: a comparative analysis. Altern Ther Health Med.

[B8] Schneider CD, Meek PM, Bell IR (2003). Development and validation of IMAQ: Integrative Medicine Attitude Questionnaire. BMC Med Educ.

[B9] NCCAM. http://nccam.nih.gov.

